# Protocol for a semi-quantitative approach to identify protein *S*-palmitoylation in cultured cells by acyl biotin exchange assay

**DOI:** 10.1016/j.xpro.2024.103054

**Published:** 2024-05-04

**Authors:** Stuart Leishman, Najd M. Aljadeed, Paras K. Anand

**Affiliations:** 1 Department of Infectious Disease, Imperial College London, London W12 0NN, UK

**Keywords:** cell biology, immunology, protein biochemistry

## Abstract

Palmitoylation is a post-translational lipid modification in which palmitic acid is conjugated predominantly to cysteine residues of target proteins, allowing them to tether to cell membranes. Here, we describe a protocol to perform a stepwise acyl biotin exchange assay to identify protein *S*-palmitoylation. We describe steps for initial blocking of free thiols in protein lysates, subsequent replacement of thioester-linked palmitate groups with a biotin tag for affinity enrichment, and identification of palmitoylated proteins by SDS-PAGE.

For complete details on the use and execution of this protocol, please refer to Leishman et al.^1^

## Highlights

• Steps for blocking free thiols in protein lysates
• Instructions for replacing thioester-linked palmitate groups with biotin tags
• Procedure to enrich biotin-tagged proteins using affinity isolation

## Abstract (editor highlights)

Publisher’s note: Undertaking any experimental protocol requires adherence to local institutional guidelines for laboratory safety and ethics.

## Graphical abstract

**Figure:**
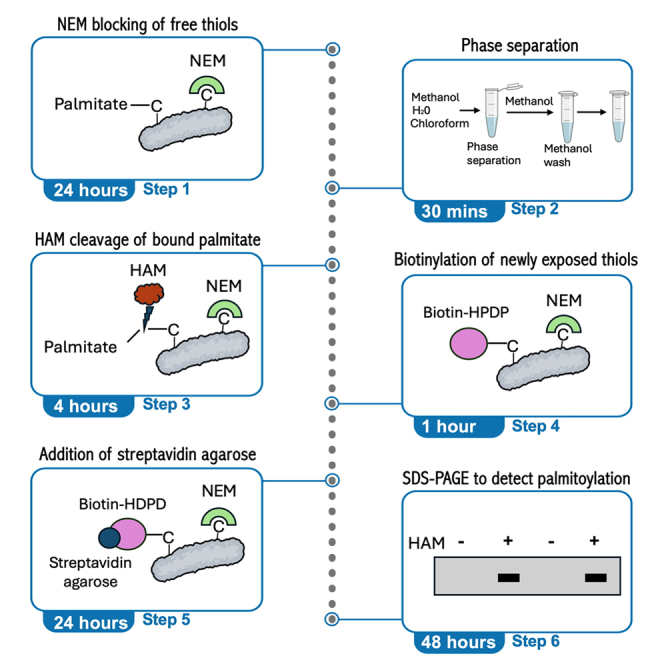

## Before you begin

Palmitoylation, or *S*-acylation, is a process whereby palmitic acid (a 16-carbon saturated fatty acid) is covalently added to cysteine residues via a labile thioester linkage.^2^ Although rare, palmitoylation can also occur on serine, threonine, or lysine residues of a protein. The attachment of palmitate increases the hydrophobicity of proteins allowing them to tether to cellular membranes.^3^ Consequently, palmitoylation is pivotal for regulating the localization, function, and stability of proteins.^4^ Some of the first proteins identified to undergo palmitoylation were the heterotrimeric G proteins and the H-Ras proteins.^5^^,^^6^ In the case of heterotrimeric G proteins, mutating the palmitoylated cysteine residues in the Gqα subunit prevented membrane attachment and activation of the downstream phospholipase C signaling pathway.^5^

In our research on NLRs and inflammasomes, we recently identified that the sensor protein NLRP3 undergoes palmitoylation.^1^ In agreement with its functions, we discovered that palmitoylation of NLRP3 promotes recruitment of the sensor protein to the *trans* Golgi network (TGN) vesicles. This translocation is important for the assembly and consequently the activation of the NLRP3 inflammasome.^7^ In cells exposed to NLRP3 stimuli such as ATP or nigericin, this membrane association triggers inflammasome signaling, resulting in the cleavage of protease caspase-1, secretion of pro-inflammatory cytokine IL-1β, and the induction of inflammatory cell death, pyroptosis.^8^^,^^9^ By contrast, inhibition of palmitoylation by a pharmacological inhibitor, 2-bromopalmitate (2-BP), results in reduced co-localization of NLRP3 with TGN vesicles and diminished caspase-1 cleavage and IL-1β secretion.^1^

Here, we describe a detailed protocol to identify palmitoylation in HEK293T cells. We strongly recommend using one of the established palmitoylated proteins as a positive control in this assay. In our protocol below, we employed Caveolin-1 as a positive control. Caveolin-1 is a major component of caveolae, membrane subdomains which are involved in organizing signaling molecules at the plasma membrane.^10^ In pulse-chase experiments conducted on endothelial cells with labeled palmitate, Caveolin-1 palmitoylation was found rather stable, making it a suitable marker for a positive control in this assay.^10^ Notably, this protocol is restricted to examining *S*-acylation in cellular lysates; it is not suitable for identifying *O*-palmitoylation or *N*-palmitoylation of proteins.^11^

### *In silico* analysis of palmitoylation sites

**Timing: 1–2 h**

This initial *in silico* analysis allows predicting putative palmitoylated sites in proteins which may assure whether the protein of interest is palmitoylated before undertaking this protocol. However, it is important to note that these predictions are not always accurate.

1. Identify the protein of interest that is to be studied. For the remainder of this protocol, NLRP3 is used as protein of interest and Caveolin-1 is employed as a control.
2. Retrieve the amino acid sequence for the protein of interest from publicly available tools such as PubMed or UniProt.
3. Input the sequence into GPS-Palm (https://gpspalm.biocuckoo.cn/) or SwissPalm (https://swisspalm.org/) which may help in the identification of putative palmitoylation residues in the protein of interest^12^^,^^13^

We performed this protocol in HEK 293T cells. HEK 293T cells do not express any of the NLR proteins. Consequently, we transfected and overexpressed Myc-tagged mouse NLRP3 in these cells. However, we have also used this protocol successfully in primary bone marrow-derived and in immortalized mouse macrophages to identify if endogenous NLRP3 undergoes palmitoylation. As such, the protocol can be used to examine palmitoylation in proteins that are sufficiently expressed endogenously in a variety of mammalian cells.

## Key resources table

**Table:** 

REAGENT or RESOURCE	SOURCE	IDENTIFIER
**Antibodies (dilution)**		

Anti-NLRP3 (1:1,000)	AdipoGen	Cat# AG-20B-0014-C100
Anti-caveolin 1 (1:1,000)	Thermo Fisher Scientific	Cat# MA3-600

**Chemicals, peptides, and recombinant proteins**		

Hydroxylamine	Sigma-Aldrich	Cat# 438227-50ML
NEM	Sigma-Aldrich	Cat# E3876-5G
Biotin-HDPD	Abcam	Cat# ab145614
Streptavidin agarose	Sigma-Aldrich	Cat# S1638-1ML
Protease inhibitor cocktail	MedChemExpress	Cat# HY-K0011
Opti-MEM	Thermo Fisher Scientific	Cat# 31985062

**Critical commercial assays**		

Pierce BCA protein assay kits	Thermo Fisher Scientific	Cat# 23225

**Experimental models: Cell lines**		

HEK 293T	ECACC	Cat# 12022001
Immortalized BMDM mouse macrophage cell line	Leishman et al.^1^	N/A

**Recombinant DNA**		

pCMV 5D Myc mouse NLRP3 plasmid	Leishman et al.^1^	N/A

**Software and algorithms**		

GPS-Palm	The CUCKOO Workgroup	https://gpspalm.biocuckoo.cn
SwissPalm	SwissPalm	https://swisspalm.org/
Image Lab	Bio-Rad	https://www.bio-rad.com/

## Materials and equipment

Buffers can be prepared in advance with the ‘core buffer’ acting as a base for lysis and flex buffers.

**Table:** Core buffer, pH 7.4 (Tris-EDTA-NaCl)

Reagent	Final concentration	Amount
Tris-HCl pH 7.4 (1.0 M)	50 mM	25.0 mL
EDTA (0.5 M)	1 mM	1.0 mL
NaCl (5.0 M)	150 mM	15.0 mL
Total	N/A	500 mL with ddH_2_O

**Table:** Lysis buffer, pH 7.4

Reagent	Final concentration	Amount
Tris-HCl pH 7.4 (1.0 M)	50 mM	0.5 mL
EDTA (0.5 M)	1 mM	0.02 mL
NaCl (5.0 M)	150 mM	0.3 mL
NP-40	1%	0.1 mL
Protease inhibitor	1 tablet/10 mL	1 tablet
Total	N/A	10.0 mL with ddH_2_O

Note: Lysis buffer can be stored at 4°C for up to 1 month.

**Table:** Flex buffer, pH range 7.0 to 8.0

Reagent	Final concentration	Amount
Tris-HCl pH 7.4 (1.0 M)	50 mM	0.5 mL
EDTA (0.5 M)	5 mM	0.1 mL
NaCl (5.0 M)	150 mM	0.3 mL
NP-40	1%	0.1 mL
SDS	1%	0.1 mL
Total	N/A	10.0 mL with ddH_2_O

Note: Flex buffer can be stored at 4°C for up to three months.

**Table:** 2X Laemmli buffer

Reagent	Final concentration	Amount
Tris base	0.125 M	0.74 *g*
SDS	0.14 M	2.0 *g*
Glycerol	20%	10.0 mL
2-mercaptoethanol	10%	5.0 mL
Bromophenol blue	N/A	100 mg
Total	N/A	50.0 mL with ddH_2_O

Note: Laemmli buffer can be stored at −20°C for over a year.

***Note:*** Stock concentrations and storage conditions of the major components in the assay are below:

### N-ethylmaleimide

Stock Concentration: 100 mM.

Final concentration: 20 mM.

Store at −20°C and use within 2 weeks of preparation.

### Hydroxylamine

Stock Concentration: 32 mM.

Final concentration: 0.7 M.

Store at room temperature.

### Biotin-HPDP

Stock Concentration: 20 mM.

Final concentration: 1.0 M.

Store at −20°C.

**CRITICAL:** N-ethylmaleimide (NEM) is toxic upon contact and fatal if swallowed. Handle under a chemical fume hood.

**CRITICAL:** NP-40 is a non-ionic surfactant. It is harmful if swallowed or inhaled and can cause skin and eye irritation. Handle under a chemical fume hood.

**CRITICAL:** 2-mercaptoethanol (2-ME) is toxic and should be handled with care.

**CRITICAL:** Sodium dodecyl sulfate (SDS) is an anionic detergent. It is harmful if swallowed and can cause skin and serious eye damage. Wear protective gloves and eye protection when handling.

***Note:*** pH of the buffers can fluctuate when stored for a long time. Make fresh buffers or check and readjust the pH, if required.

## Step-by-step method details

### Transfection and stimulation of HEK 293T cells

**Timing: 2 days**

In this step, cells are transfected with Myc-NLRP3 plasmid to over-express the NLRP3 protein.

#### Day 0

1. Plate ∼2 × 10^6^ HEK 293T cells per well in a 6-well tissue culture plate.
2. Each confluent 6-well yields ∼200 μg of protein which should be sufficient for generating HAM positive, and HAM negative control samples later in the protocol.
  a. We recommend using a minimum of 100 μg protein per sample.
  b. The plating density may need to be optimized for other cell types so that the wells are approximately 90% confluent.
3. Allow cells to grow in a 37°C incubator for 18 h–24 h.

#### Day 1

4. Change media to Opti-MEM to get cells ready for transfection.
  a. Transfection can be omitted if the protein of interest is expressed well in HEK 293T cells or in your chosen cell type.
5. Add 1.5 μL Lipofectamine 2000 in a final volume of 25 μL OptiMEM in a 1.5 mL tube. Vortex gently for ∼10 s.
6. In a separate 1.5 mL tube, add plasmid DNA in a final volume of 25 μL OptiMEM. Vortex gently for ∼10 s.
  a. In this example, Myc-NLRP3 plasmid was transfected at 0.5 μg/well in a 6 well plate.
  b. If several wells are to be transfected, the above reagents can be prepared as a master mix.
7. Let the two tubes sit for 5 min at 22°C–24°C.
8. Mix the contents of the two tubes into a single tube and gently vortex for ∼10 s. Incubate the tube containing both Lipofectamine 2000 and plasmid DNA for 25 min at 22°C–24°C.
9. Add 50 μL complex to the desired wells dropwise.
10. Incubate the cells for 18 h–24 h in a 37°C incubator.
  a. Depending on the plasmid and the gene of interest, the cells may take between 24 h and 72 h to express the gene of interest. This may need to be optimized in a pilot experiment examining the protein expression at different time points.

#### Day 2

11. On the day of experiment, wash the cells once with PBS and change growth media to fresh 1 mL DMEM.
12. Activate the inflammasome with a final concentration of 5 mM ATP for 30 min.
  a. As HEK 293T cells do not express the full suite of inflammasome components, they do not undergo pyroptosis.
  b. Primary and immortalized mouse macrophages will first need to be stimulated with LPS (500 ng/mL) for 4 h in a 37°C incubator to allow cells to express inflammasome components. Note that these cells rapidly undergo pyroptosis upon inflammasome activation which may affect protein yield between samples.
  c. It is important to note that palmitoylation may or may not require cell stimulation. This may depend on the expression of specific palmitoyl acyltransferases.
13. Using a cell scraper, collect cells in a sterile 1.5 mL Eppendorf tube.
14. Spin down the cells in a microcentrifuge for 5 min at 1,000 *g* at RT.
15. Wash the cell pellet in 1 mL PBS.

### Acyl-biotin exchange (ABE) for identification of *S*-acylated sites

**Timing: 2–3 days**

### N-ethylmaleimide blocking of free thiol sites

**Timing: 1 day**

In this step, N-ethylmaleimide (NEM), an alkene derived from maleic acid, is added to irreversibly block free (unbound) thiol group of cysteine residues. This step ensures that only cysteines that were originally palmitoylated are examined in the later steps of this protocol.

16. Resuspend the cell pellet from the previous step in 200 μL lysis buffer containing 20 mM NEM.
17. Vortex 3 times at 3 s intervals.

**CRITICAL:** pH of the lysis buffer is crucial for NEM to function and should be readjusted if the buffer was stored for a long time. The pH should be between 7.0 and 8.0 for optimal NEM function.

18. Incubate samples at 4°C for approximately 16 h–18 h with end-over-end rotation.
19. Precipitate the protein with a mixture of chloroform, methanol, and water at a ratio of 1:0.4:1 (methanol: chloroform: H_2_O).
  a. For the initial 2 × 10^6^ cells used, the volumes we add are 500 μL methanol, 200 μL chloroform, and 300 μL H_2_O. 200 μL of lysis buffer contributes to the aqueous part.
20. Vortex the samples for ∼10 s, and centrifuge at 15,000 *g* for 5 min.
21. The centrifugation step should result in a phase separation resulting in three distinct layers (Figure 1A). With a p1000 pipette, remove the topmost aqueous layer making sure not to disturb the middle interphase consisting of proteins.
  a. If the aqueous layer cannot be completely removed without disturbing the protein interphase, it can be left for removal in the subsequent wash steps.

**Figure 1:**
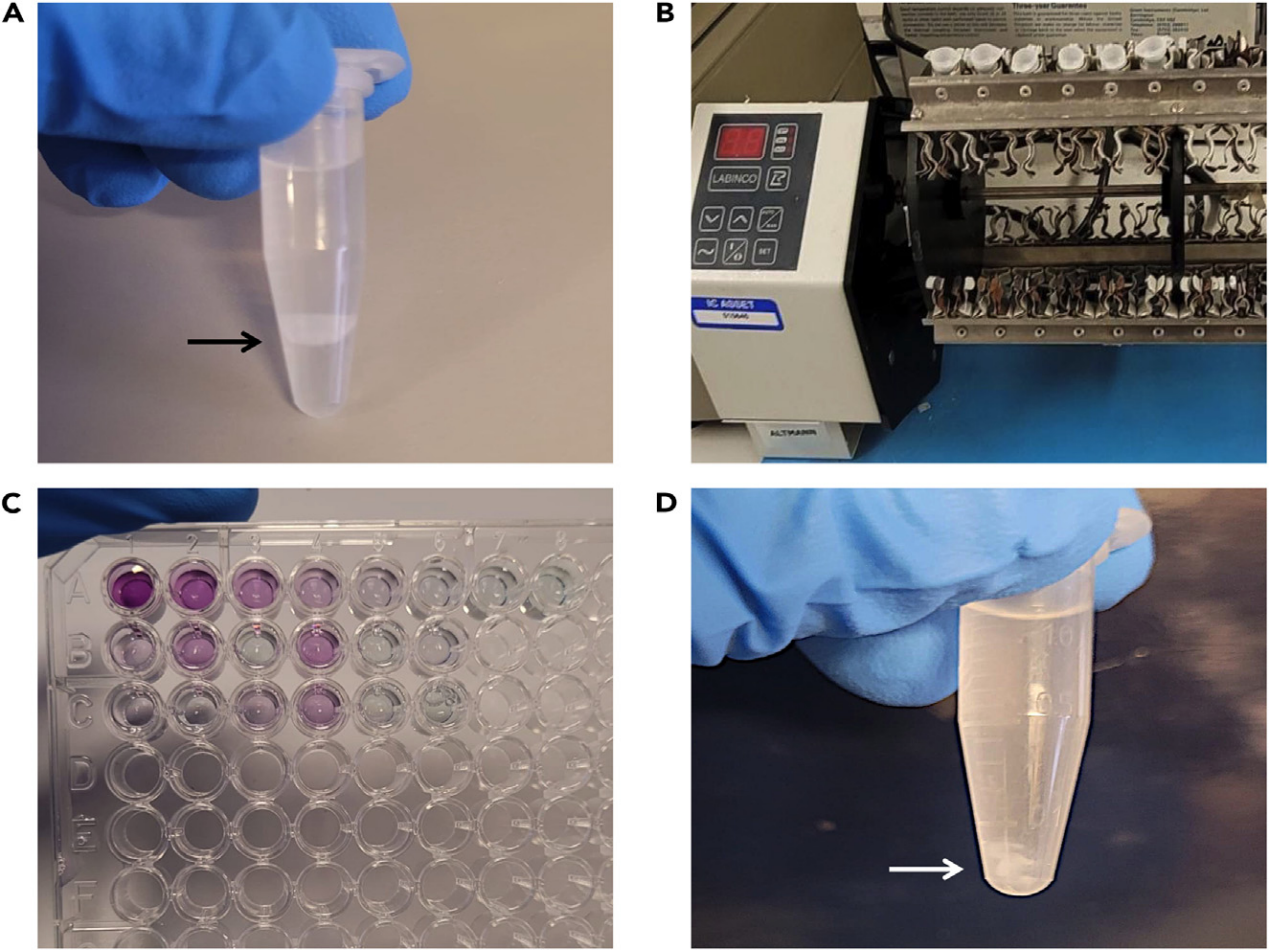
Images depicting some of the steps of the acyl-biotin exchange (ABE) assay (A) Protein precipitation: clear layers can be observed, with the upper phase (metabolites, salts, and amino acids), middle protein interphase shown with an arrow, and lower phase consisting of lipids. (B) Example of end-over-end rotation of samples to ensure proper reagent mixing. (C) Color development in BCA assay for protein quantification. (D) Affinity enrichment of palmitoylated proteins by streptavidin agarose. Note the protein pellet at the bottom of the tube shown with an arrow.
22. Add 500 μL of methanol and mix by inverting the tubes 3–5 times.
23. Spin at 15,000 *g* for 5 min in a microcentrifuge. This should result in a relatively stable pellet at the bottom of the tube.
24. Carefully decant or remove the supernatant with a pipette tip.
25. Repeat protein precipitation steps 19–24 for an additional 2x times.
26. Leave the pellets to air dry for 10 min.

**CRITICAL:** Complete removal of residual NEM by repeating protein precipitation steps is important to prevent further recapping of the free thiol groups made available by cleavage of the thioester bond that linked palmitic acid to cysteine residues. Any left-over NEM in the samples will lead to a reduction or complete loss of palmitoylation signal.

**Pause point:** Samples can be stored at −20°C for up to 24 h. Leaving the samples for long-term at this step is not recommended.

### Cleavage of palmitate-bound thiol sites by hydroxylamine (HAM)

**Timing: 3–4 h**

Addition of HAM in this step cleaves the thioester bond between the sulfhydryl group of cysteine residue and the palmitic acid. This newly available unbound (free) cysteine sulfhydryl group is used for biotin tagging. Hydroxylamine is not able to cleave the NEM bound sites, and these remain unavailable to the biotin tag.

27. Resuspend the protein pellet in 400 μL of flex buffer. Vortex to dissolve the protein pellet. Brief sonication up to ∼30 s may be required.
28. Once the protein pellet is fully dissolved in the cleavage buffer, divide the samples equally (200 μL each) into pre-labeled HAM (−) and HAM (+) tubes.
29. Add 4.5 μL HAM (0.7 M final concentration) to tubes labeled HAM (+).
30. Incubate with end-over-end rotation (Figure 1B) at 22°C–24°C for 1 h.
31. Finally, perform chloroform: methanol protein precipitation one time as described in steps 19–24.
  a. Allow pellets to dry fully before proceeding further.

### Thiol site biotinylation and streptavidin-agarose pull-down

**Timing: 1 day**

In this step, biotin tag is added to the newly available free thiol sites which were previously palmitoylated for affinity enrichment. The palmitoylated proteins can be detected using antibody against the protein of interest or if the protein was co-expressed with a tag on it.

32. Dissolve the protein pellet in 200 μL of flex buffer supplemented with 20 mM Biotin-HPDP.
33. Incubate the samples at 22°C–24°C with end-over-end rotation for 1 h.
34. Perform protein precipitation one final time as described in steps 19–24.
35. Dissolve the protein in 100 μL of flex buffer.
36. Estimate protein concentration by mini-BCA assay (Figure 1C) using a 10 μL protein sample.
37. Normalize protein concentration for any variability across samples.
  a. Remove a 20 μL aliquot from each sample to load as an input control. Mix the sample with 20 μL of 2x SDS-PAGE Laemmli buffer and store at −20°C until ready to load.
38. Dilute the remaining 70 μL sample 10 times by adding 630 μL of core buffer. This dilutes the SDS concentration to 0.1%.
39. Add 30 μL of pre-washed streptavidin agarose beads directly to each sample and vortex for ∼10 s to mix the contents.
  a. To first wash and prepare the streptavidin-agarose beads, add 30 μL agarose beads per sample to 500 μL of core buffer.
  b. Spin at 10,000 g for 2 min at room temperature.
  c. Wash the agarose beads for a total of 3 times taking 500 μL core buffer each time. After the final wash step, resuspend the beads in 30 μL of core buffer per sample.
40. Incubate the samples with end-over-end rotation at 4°C for 18 h–24 h.
41. The following day, wash the samples with 500 μL of core buffer supplemented with 0.1% SDS and 0.1% NP-40.
  a. Repeat washes for a total of 3 times, spinning the samples at 12,000 *g* for 5 min between each of the washes.
42. To the pellet (Figure 1D), add 75 μL of 1x Laemmli buffer and store at −20°C until ready for SDS-PAGE.

## Expected outcomes

**Figure 2:**
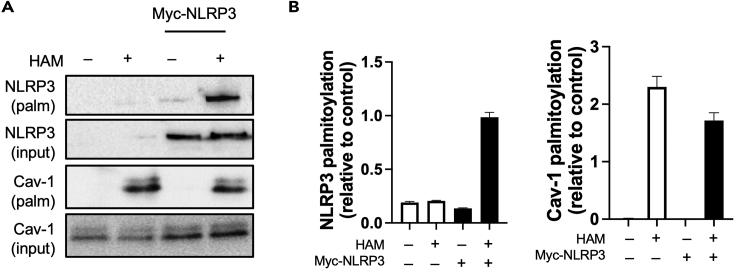
Expected outcomes from an acyl-biotin exchange (ABE) assay (A) HEK-293T cells were transfected with Myc-NLRP3 and an ABE assay was carried out. The samples and input controls were immunoblotted with either NLRP3 or Caveolin-1 antibody. Note the absence (or very low expression) of the two proteins in HAM (−) samples compared to HAM (+) samples. (B) Densitometric analysis of the blot shown in (A) to illustrate the semi-quantitative nature of this protocol. Data shown are mean with error bars showing standard deviation, and are representative of experiments done at least three times.

When this protocol is followed for a protein which has active palmitoylation sites at the time of sample collection, it can be expected to observe a band only in the HAM (+) set of samples after immunoblotting. A band should not be observed in the corresponding HAM (−) samples. Representative blots for our protein of interest NLRP3 and Caveolin-1 are shown in Figure 2A. The corresponding input control samples should also be run for immunoblotting. This allows for normalization of data and more accurate comparison between different streptavidin pull-down samples or different experimental conditions. When combined with standard densitometric analysis of immunoblots such as with ImageJ (NIH) or Image Lab (Bio-Rad), it is possible to obtain semi-quantitative data from these blots, as shown in Figure 2B. If the assay has proceeded expectedly as evaluated from the data obtained with controls in the experiment, but no bands have been observed in HAM (+) samples, it can be concluded that the protein is not *S*-palmitoylated.

## Limitations

Whilst robust, there are limitations to this assay. The method can only detect cysteine palmitoylation (*S*-palmitoylation). Palmitoylation at serine, threonine or lysine residues cannot be detected with this assay. The assay involves multiple steps, and proteins which are rather unstable or insoluble, may be degraded or lost during the protocol giving misleading results. The assay is also less sensitive to detect proteins with limited palmitoylation or those which are expressed at low abundance in samples. Finally, the assay has quantitative limitations. It cannot quantify the number of active palmitoylated sites. But useful semi-quantitative information on the levels of palmitoylation between different experimental conditions can be obtained by using this protocol.

## Troubleshooting

### Problem 1

Smearing or fragmentation of protein bands (related to steps 2 and 41).

### Potential solution

Smearing on the gel can either be due to the presence of agarose beads in the sample when loading the samples, or higher protein concentration of the samples. This can be minimized by ensuring that samples are centrifuged to remove the agarose beads after all the bound protein has been recovered following boiling of the samples. Protein loading may need to be optimized.

### Problem 2

Low palmitoylation signal in HAM (+) samples (related to step 25).

### Potential solution

This can be due to the protein having no active palmitoylation. It can also be due to protein having low palmitoylation levels which are below the sensitivity levels of this assay. However, if palmitoylation of the protein is known, then this could be due to the residual levels of NEM in the sample. Make sure that NEM is completely removed before proceeding to the addition of HAM to the samples. This can be done by increasing the number of protein precipitation steps after overnight incubation with NEM. If the problem still remains, NEM concentration in the buffer can be lowered to 15 μM but this may affect the specificity of the assay.

### Problem 3

**Figure 3:**
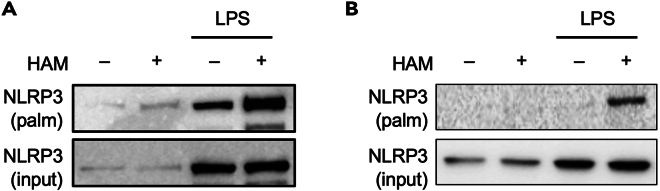
Examples of immunoblots depicting a bad vs. good outcome in an acyl-biotin exchange (ABE) assay Immunoblots were carried out on immortalized BMDMs exposed or not to LPS (500 ng/mL). Upper panels show palmitoylated NLRP3 while lower panels show NLRP3 immunoblot on input samples. The protein shows only low constitutive expression which is upregulated upon TLR4 ligation by LPS. Panel (A) shows a bad outcome in which HAM (−) sample exhibits high non-specific pull-down of NLRP3. Panel (B) depicts a good outcome with minimal NLRP3 pull-down in HAM (−) sample compared to a strong band observed in HAM (+) sample. Please refer to recommendations under ‘troubleshooting’ to achieve a good outcome of this protocol.

Presence of non-specific bands in HAM (−) samples (related to step 34 and Figure 3).

### Potential solution

If the total amount of starting protein is too high, NEM may not efficiently block free thiol sites leading to non-specific biotinylation and consequently pull-down by streptavidin agarose. This may result in bands in the HAM (−) samples. An increased abundance of biotin in the samples may also lead to the above effect. Keep the starting protein concentration around the recommended range. Add an extra protein precipitation step to remove excess unbound biotin. Figure 3A shows a bad outcome of this protocol in which comparable bands are observed in HAM (+) and HAM (−) samples. Figure 3B shows a good outcome of the protocol illustrating no non-specific pull-down of NLRP3 in HAM (−) sample after the above troubleshooting steps have been taken.

### Problem 4

Difficulties in dissolving the protein pellet after protein precipitation steps (related to step 27).

### Potential solution

If the pellet will not dissolve, then it indicates the presence of methanol in the samples. This is a common occurrence and may require vigorous vortexing or physically breaking the pellet with a micro spatula. A few seconds in bath sonication may also help. Try and keep loss of protein minimal during the resuspension.

### Problem 5

Complete absence of protein on gels.

### Potential solution

Do a rigorous check of all molecular biology reagents to ensure that plasmid transfection of HEK 293T cells worked. Also, use a fresh aliquot of the antibody used in immunoblotting.

## Resource availability

### Lead contact

Further information and request for resources and reagents should be directed to and will be fulfilled by the lead contact, Paras Anand (paras.anand@imperial.ac.uk).

### Technical contact

Further information and technical help should be directed and fulfilled by the technical contact, Stuart Leishman (s.leishman21@imperial.ac.uk).

### Materials availability

This study did not generate any new unique reagents.

### Data and code availability

This study did not generate any new datasets/code.
