# Protocol for a semi-quantitative approach to identify protein *S*-palmitoylation in cultured cells by acyl biotin exchange assay

**DOI:** 10.1016/j.xpro.2026.104638

**Published:** 2026-06-10

**Authors:** Stuart Leishman, Najd M. Aljadeed, Paras K. Anand

## Main text

(STAR Protocols *5*, 103054; June 21, 2024)

During manuscript preparation, two concentration volumes were inadvertently misreported in the “materials and equipment” and “step-by-step method details” sections of this protocol.

• Under “materials and equipment,” (1) the stock concentration for hydroxylamine reads “32 mM” while it should read “32 M,” and (2) the final concentration for biotin-HPDP reads “1.0 M” while it should read “1.0 mM.”
• In step 32 of “step-by-step method details,” the “20 mM Biotin-HPDP” that is mentioned should instead read “Biotin-HPDP to 1 mM final concentration” due to the above changes.

The authors apologize for the errors and regret any confusion caused.

